# The Impact of Maternal Smoking During Pregnancy on Depressive and Anxiety Behaviors in Offspring: A Meta-analysis

**DOI:** 10.1155/da/2168791

**Published:** 2025-08-26

**Authors:** Kui Zhang, Yu Wang, Zhilong Shu, Ying Huang, Lixiang Feng, Wenxing Yang

**Affiliations:** ^1^Department of Forensic Pathology, West China School of Basic Medical Sciences and Forensic Medicine, Sichuan University, Chengdu, Sichuan 610041, China; ^2^Department of Physiology, West China School of Basic Medical Sciences and Forensic Medicine, Sichuan University, Chengdu, Sichuan 610041, China

**Keywords:** anxiety, depression, maternal smoking, meta-analysis, pregnancy

## Abstract

Smoking during pregnancy is known to adversely affect offspring health; however, the association between maternal smoking during pregnancy and the risk of depression and anxiety in offspring remains inconsistent. This meta-analysis aimed to clarify this relationship. A systematic search was conducted in PubMed, Web of Science, and OVID databases for articles published between 2000 and 2024. The odds ratio (OR) with a 95% confidence interval (CI) was used to assess the association. A total of 11 studies involving 1,775,220 participants met the inclusion criteria. The meta-analysis revealed that maternal smoking during pregnancy was significantly associated with an increased risk of depression in offspring (OR = 1.33, 95% CI = 1.09–1.63). Stratified analysis by cigaret consumption dose showed that heavy maternal smoking (≥ 10 cigarets/day) further increased the risk of both depression (OR = 1.61, 95% CI = 1.21–2.14) and anxiety (OR = 1.51, 95% CI = 1.32–1.72) in offspring. In conclusion, this meta-analysis provides evidence that maternal smoking during pregnancy may elevate the risk of depression and anxiety in offspring, particularly with heavy smoking. Preventing maternal smoking and reducing exposure to tobacco smoke during pregnancy could have significant benefits for offspring mental health and well-being.

**Summary**

• Maternal smoking during pregnancy is widely recognized for its potential adverse health effects on offspring. However, the relationship between prenatal maternal smoking and the risk of depression and anxiety in children remains a topic of debate.
• Our meta-analysis, which included 11 studies with a total of 1,775,220 participants, aimed to clarify the association between maternal smoking during pregnancy and the risk of depression and anxiety in offspring.
• The results of our analysis indicated that prenatal maternal smoking is significantly associated with an increased risk of depression in offspring, stratified analysis further revealed that heavy maternal cigaret consumption during pregnancy (≥ 10 cigarets per day) was linked to elevated risks of both depression and anxiety in children.
• Interestingly, our study did not observe a significant association between partner smoking during pregnancy and the risk of depression in offspring.

## 1. Introduction

Tobacco use is projected to result in up to 1 billion deaths worldwide in the 21^st^ century, primarily due to noncommunicable diseases [1]. Globally, 40% of children, 33% of male nonsmokers, and 35% of female nonsmokers are exposed to secondhand smoke, which remains a major cause of morbidity and mortality [2].

Maternal smoking during pregnancy is a significant public health concern, with a globe prevalence of approximately 1.7% [3, 4], and 2.6% in low-income and middle-income countries [1, 5]. However, prevalence rates vary significantly across regions. Maternal smoking during pregnancy poses severe risks to both the mother and the child, including adverse developmental, physical, and psychological outcomes [6–11].

Mental disorders are increasingly recognized as major contributors to the global disease burden [12], with depression and anxiety being the most prevalent conditions [13, 14]. These two disorders collectively account for 8% of years lived with disability worldwide [15]. Among mental disorders, depressive disorders contribute the most to disability-adjusted life years, followed closely by anxiety disorders. Notably, depression and anxiety often co-occur: upto 25% of general practice patients experience both conditions, and approximately 85% of individuals with depression report significant anxiety, and 90% of those with anxiety also experience depression [16].

In addition to genetic predisposition, several environmental and psychosocial factors, such as smoking, unplanned pregnancy, lack of social support, alcohol use, poor sleep quality, academic stress, unemployment, and financial difficulties, can significantly increase the risk of depression and anxiety [17]. Recent evidence also highlights the role of non-DNA sequence-based epigenetic inheritance, which can influence multiple generations in organisms ranging from yeast to humans [18].

Maternal smoking during pregnancy has been widely reported to adversely affect the health of offspring [19–22]. However, the relationship between maternal smoking during pregnancy and the risk of depression and anxiety in offspring remains unclear. To address this gap, we conducted a comprehensive meta-analysis of published studies to quantify the association between maternal smoking during pregnancy and the risk of depression and anxiety behaviors in offspring. This study adheres to the preferred reporting items for systematic reviews and meta-analyses (PRISMA) guidelines [23].

## 2. Methods

### 2.1. Publication Search and Inclusion Criteria

**Figure 1:**
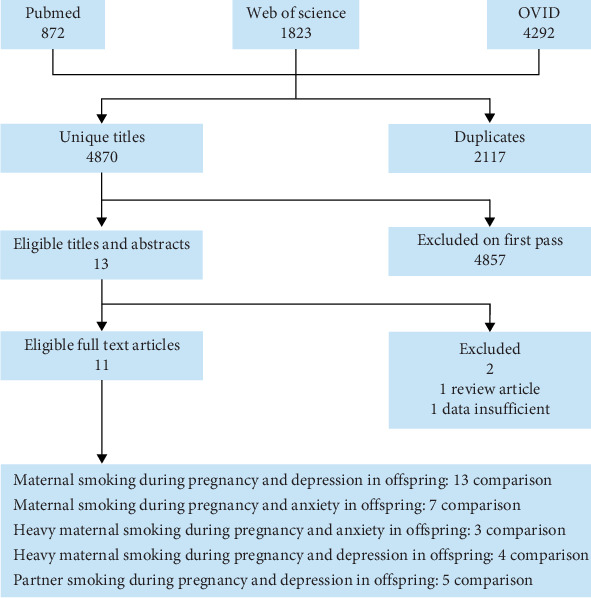
Flowchart for identification of studies.

We performed an extensive search across the PubMed, Web of Science, and OVID databases for studies published between 2000 and 2024 that examined the relationship between maternal smoking during pregnancy and depressive and anxiety behaviors in offspring (with the last search update on June 10, 2024). The search strategy included the following terms: “maternal” AND “smoking” AND “pregnancy” AND '“depression” or “anxiety” AND “children” or “offspring”. Additionally, references from the selected studies were reviewed to identify further relevant publications. This search resulted in a total of 4870 abstracts (Figure 1). The quality of cohort and case–control studies was assessed using the Newcastle–Ottawa Scale (NOS), which considers three categories and eight items.

The criteria for including studies were: (1) examination of the relationship between maternal smoking during pregnancy and depressive and/or anxiety behaviors in offspring; (2) use of the generalized anxiety disorder 7-item (GAD-7) scale for anxiety, the patient health questionnaire (PHQ-9) for depression, or the self-reporting questionnaire 20-Item (SRQ-20) scale for any common mental disorder; (3) availability of odds ratios (ORs) and 95% confidence intervals (CIs) in the manuscript or supporting information, or sufficient data to calculate these metrics; (4) classification as a cohort or case-control study; and (5) publication in English.

### 2.2. Data Extraction

Two authors (Kui Zhang and Yu Wang) independently reviewed and extracted the required data. Discrepancies were resolved through discussions among the authors to reach a consensus. The recorded information for each study included: first author, year of publication, region, follow-up period, type of mental illness, participants, and confounding factors, such as gender and family history of depression or anxiety (Table 1).

### 2.3. Statistical Analysis

To examine the potential link between maternal smoking during pregnancy and depressive or anxiety behaviors in offspring, we employed the odds ratio (OR) alongside the 95% confidence interval (95% confidence interval [CI]). Our meta-analysis focused on published studies, aiming to estimate the risk of depressive or anxiety outcomes in offspring attributed to maternal smoking during pregnancy. Furthermore, we explored the influence of heavy cigaret consumption on these outcomes. In addition, we investigated the association between partner smoking during pregnancy and the risk of depression in offspring.

Statistical heterogeneity among the studies was evaluated utilizing the *Q*-test and *I*^2^ statistics [35]. Depending on the heterogeneity, either a fixed-effects or random-effects model was employed to estimate the overall OR [36, 37]. OR estimates and their 95% CIs were calculated using random effects models. To identify potential sources of heterogeneity across the studies, logistic meta-regression analyses were conducted. The study characteristics examined included publication year, geographical region, follow-up duration, number of cases, number of controls, and morbidity. Subgroup analyses by sample size and gender distribution were also performed. Publication bias was assessed with a funnel plot and Begg's rank correlation method [38]. All statistical analyses were performed using STATA 17.0 software (Stata Corp., College Station, TX).

## 3. Results

### 3.1. Characteristics of Studies

Out of a total of 4870 abstracts screened, 13 were selected for more detailed evaluation. One paper was excluded as it was a review, and one was excluded due to insufficient data (shown in Figure 1). Ultimately, 11 studies met the inclusion criteria for this analysis [24–34], with 1,775,220 participants. The details of included studies were listed in Table 1.

### 3.2. Quantitative Synthesis

**Figure 2:**
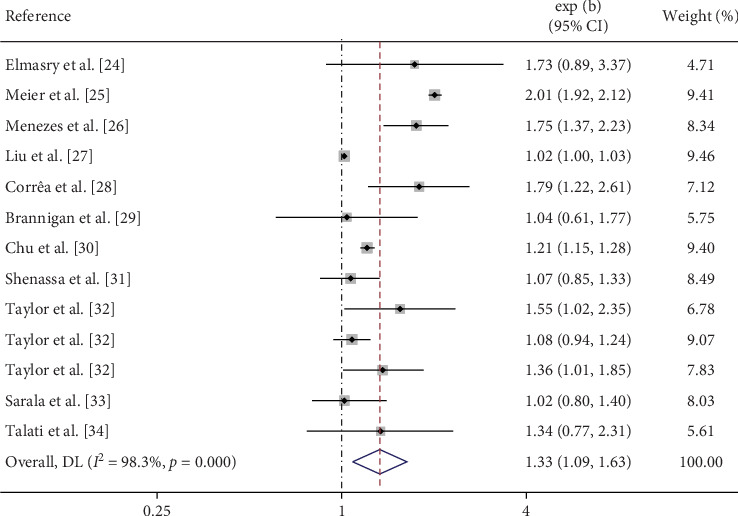
Forest plot of depressive risk associated with maternal smoking during pregnancy.

**Figure 3:**
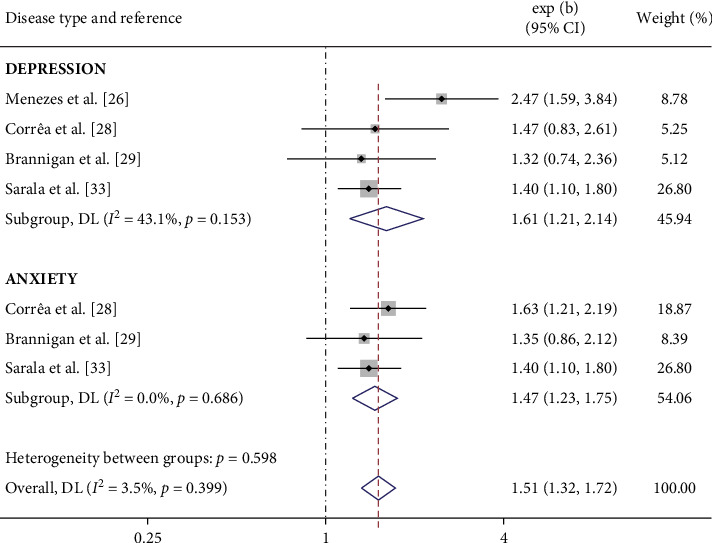
Forest plot of depression and anxiety risk associated with heavy maternal cigaret consumption during pregnancy.

**Figure 4:**
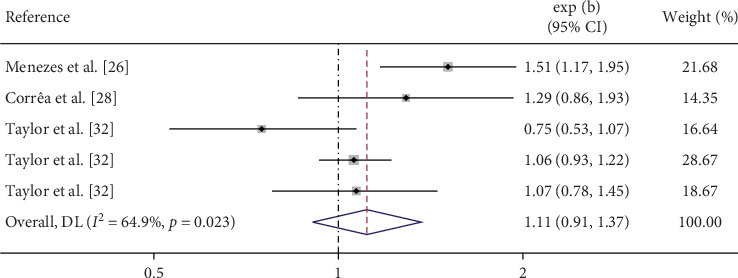
Forest plot of depression risk associated with partner smoking during pregnancy.

The accumulated data from this meta-analysis indicate that maternal smoking during pregnancy increases the risk of depression in offspring (OR = 1.33, 95% CI = 1.09–1.63, Figure 2). However, no significant association was observed between maternal smoking during pregnancy and anxiety in offspring (OR = 1.28, 95% CI = 0.98–1.67). In the stratified analysis, heavy maternal cigaret consumption during pregnancy was associated with an increased risk of both depression (OR = 1.61, 95% CI = 1.21–2.14) and anxiety (OR = 1.51, 95% CI = 1.32–1.72) in offspring (Figure 3). Additionally, no significant association was observed between partner smoking during pregnancy and depression in offspring (Figure 4).

### 3.3. Evaluation of Heterogeneity

Significant heterogeneity was observed among studies in the overall comparisons (*p* heterogeneity < 0.001, *I*^2^ = 98.3% for depression; *p* heterogeneity < 0.001, *I*^2^ = 99.3% for anxiety). To identify the sources of heterogeneity, we performed meta-regression to assess variables, such as publication year, region, follow-up period, disease type, and sample size. Finally, we found that the sample size may substantially impacted the initial heterogeneity (*I*^2^ = 56.0%). Subgroup analysis by sample size revealed stable results (OR = 1.26, 95% CI = 1.07–1.47 for sample size ≤ 3000 and OR = 1.32, 95% CI = 1.02–1.70 for sample size > 3000).

### 3.4. Sensitivity Analysis

The influence of individual studies on the overall meta-analysis estimate was assessed by sequentially omitting each study. The omission of any single study did not significantly alter the results for depression, indicating the statistical robustness of the findings. No significant association was observed between maternal smoking during pregnancy and anxiety in offspring (OR = 1.28, 95% CI = 0.98–1.67). Sensitive analysis by omitting of the studies by Liu L [27] and Talati A [34] significantly affected the results (OR = 1.41, 95% CI = 1.16–1.71 for omitting the studies by Liu L [27] and OR = 1.36, 95% CI = 1.03–1.80 for omitting the studies by Talati A [34]). This result highlights the instability of the findings on maternal smoking during pregnancy and its association with anxiety in offspring. Given that the overall CI (0.98–1.67) is close to the threshold for statistical significance, there is a possibility that the observed lack of association is a false negative. Further research is needed to clarify this potential false-negative outcome.

### 3.5. Publication Bias

**Figure 5:**
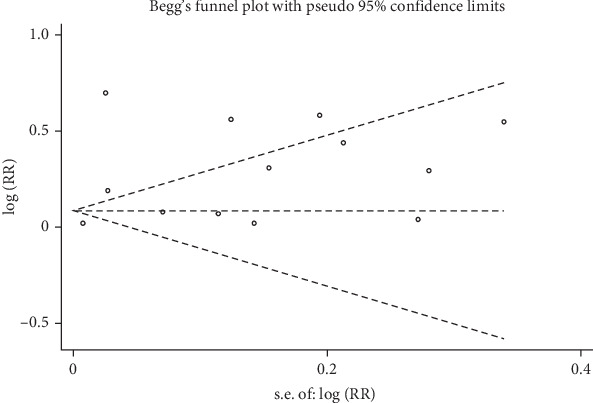
Begg's funnel plot for publication bias test. Each point represents a separate study for the indicated association. s.e., standardized effect.

The Begg's test was performed to evaluate publication bias among the selected studies. No evidence of publication bias was observed (*p*=0.669 for depression and *p*=0.368 for anxiety). Begg's funnel plots for the publication bias test have been included as Figure 5.

## 4. Discussion

This meta-analysis examined 11 published studies involving 1,775,220 participants to evaluate the association between maternal smoking during pregnancy and the risk of depression and anxiety in offspring. The results demonstrated a significant increase in the risk of depression among offspring exposed to maternal smoking during pregnancy. Notably, heavy maternal smoking, defined as consuming ≥10 cigarets per day, was found to increase the risk of depression and anxiety in offspring.

Depression and anxiety are commonly categorized as internalizing disorders that exhibit overlapping yet distinct symptom profiles [39]. These internalizing behaviors have been linked to disruptions in cortico-amygdalar networks, including the parieto-amygdalar network [40], the default mode network [41], and the fronto-amygdalar network [42]. Maternal smoking during pregnancy can interfere with neurodevelopment, affecting critical neurotransmitter systems and brain regions responsible for stress regulation and mood processing [43, 44]. Thus, maternal smoking represents a modifiable risk factor for maladaptive emotional development in offspring [26].

Our findings are consistent with previous research demonstrating a significant association between maternal smoking during pregnancy and an increased risk of depression in offspring [25, 26, 30]. Although, several studies have also reported a positive association between maternal smoking and the risk of anxiety disorders [25, 28, 30, 33], our results did not support this relationship. Notably, the CI (0.98–1.67) for this association was close to the threshold of statistical significance, suggesting potential instability. Sensitivity analysis further revealed that omitting either of two studies [27, 34] could shift the association toward statistical significance. These data highlight the fragility of the observed results and the need for cautious interpretation. Experimental evidence demonstrates, that nicotine exposure can induce anxiety-like behaviors in animal models. However, its long-term effects on offspring in humans remain inconclusive and appear to be context-dependent [45, 46]. This inconsistency may contribute to the observed ambiguity regarding the association between maternal smoking during pregnancy and the risk of anxiety disorders in offspring. Collectively, these findings suggest that the relationship between maternal smoking during pregnancy and the risk of anxiety in offspring remains uncertain. To address this question more conclusively, further research is needed, particularly studies with larger datasets, robust analytical methods, and a focus on minimizing potential confounding factors.

Secondhand smoke remains a major public health concern, with studies indicating substantial exposure levels among nonsmokers and adolescents [47, 48]. While, nicotine exposure through passive smoking has been shown to influence neurotransmitter systems and brain development [49, 50], the evidence linking secondhand smoke to mental health outcomes remains ambiguous [48, 51–53]. Interestingly, our study did not observe a significant association between partner smoking (a kind of secondhand smoking) and these outcomes, several reasons may help to explain it. First, partners may consciously limit smoking behaviors during pregnancy due to increasing awareness of the risks associated with secondhand smoke exposure. For example, partners may avoid smoking indoors or near pregnant women, thereby minimizing the extent of secondhand smoke exposure, such behavioral changes could reduce the measurable impact of partner smoking on offspring outcomes. Second, there is a notable scarcity of studies specifically examining the effects of partner smoking on offspring mental health outcomes, most existing evidence on secondhand smoke focuses on respiratory health and birth weight rather than long-term behavioral or psychological effects. Consequently, the current body of literature may lack the statistical power and depth to establish significant associations. Finally, maternal smoking exposes the fetus directly to nicotine and other harmful metabolites via placental transfer. In contrast, partner smoking primarily contributes to secondhand smoke exposure, which indirectly affects the mother and, subsequently, the fetus. The differences in exposure pathways and magnitude may account for the weaker or null association observed in our study.

Several limitations of this study should be considered. First, although publication bias was not detected, its presence cannot be entirely excluded in any meta-analysis. Second, sensitivity analysis revealed instability in risk estimates for anxiety disorders, highlighting the need for more robust data to strengthen these findings. Third, due to the limited number of available studies, certain confounding factors—such as family history of depression/anxiety, socioeconomic status, and maternal mental health—could not be thoroughly examined. Future studies with larger sample sizes and more detailed study designs are needed to address these gaps.

## 5. Conclusion

In conclusion, this meta-analysis provides evidence that maternal smoking during pregnancy is associated with an increased risk of depression in offspring, preventing maternal smoking and reducing exposure to tobacco smoke during pregnancy may have significant benefits for offspring mental health. Future research should focus on elucidating the underlying mechanisms, addressing potential confounding factors, and further exploring the association with anxiety disorders to strengthen the evidence base for preventive interventions.

## Figures and Tables

**Table 1:** Characteristics of literature included in the meta-analysis.

Reference	Year	Region	Follow-up time (year)	Heavy smoking (cigarets/day)	Disease	Number of participates	Depression patients	Anxiety patients	Female (%)
Elmasry et al. [24]	2014	USA	7.00	—	Depression	176	44	—	NA
Meier et al. [25]	2017	Denmark	5.00	—	Depression and anxiety	957,635	6525	6739	48.9
Menezes et al.[26]	2013	Brazil	11.00	≥20	Depression	4106	282	—	50.9
Liu et al.[27]	2023	UK	Adults	—	Depression, anxiety	342,846	110,948	98,702	NA
Corrêa et al. [28]	2022	Brazil	22	≥10	Depression and anxiety	3780	110	396	53.4
Brannigan et al.[29]	2022	Ireland	30.00	≥5	Depression and anxiety	3619	NA	NA	NA
Chu et al.[30]	2021	UK	Adults	—	Depression and anxiety	432,881	6277	15,385	53.3
Shenassa et al. [31]	2023	USA	Adults	—	Depression	1692	397	—	57.1
Taylor et al. [32]	2017	UK	17.00	—	Depression	2869	198	—	55.2
Taylor et al. [32]	2017	Norway	32.00	—	Depression	15,493	1208	—	52.1
Taylor et al. [32]	2017	Brazil	30.00	—	Depression	2626	202	—	51.6
Sarala et al. [33]	2022	Finland	29.00	≥10	Depression and anxiety	7259	NA	672	50.5
Talatiet al. [34]	2017	USA	27.7	—	Depression and anxiety	238	128	170	49.0

## Data Availability

The data used to support the findings of this study are available from the corresponding author upon request.
